# Glutamate, Humic Acids and Their Combination Modulate the Phenolic Profile, Antioxidant Traits, and Enzyme-Inhibition Properties in Lettuce

**DOI:** 10.3390/plants12091822

**Published:** 2023-04-28

**Authors:** Marco Armando De Gregorio, Gökhan Zengin, Fatma Nur Alp-Turgut, Fevzi Elbasan, Ceyda Ozfidan-Konakci, Busra Arikan, Evren Yildiztugay, Leilei Zhang, Luigi Lucini

**Affiliations:** 1 Department of Sustainable Food Process, Università Cattolica del Sacro Cuore, 29122 Piacenza, Italy; marcoarmando.degregorio@unicatt.it (M.A.D.G.);; 2 Department of Biology, Faculty of Science, Selcuk University, Selcuklu, 42130 Konya, Turkey; 3 Department of Biotechnology, Faculty of Science, Selcuk University, Selcuklu, 42130 Konya, Turkey; 4 Department of Molecular Biology and Genetics, Faculty of Science, Necmettin Erbakan University, Meram, 42090 Konya, Turkey; 5 CRAST Research Centre, Università Cattolica del Sacro Cuore, 29122 Piacenza, Italy

**Keywords:** lettuce, antioxidant, metabolomics, enzyme inhibition, functional applications

## Abstract

Lettuce (*Lactuca sativa* L., Asteraceae) is a popular vegetable leafy crop playing a relevant role in human nutrition. Nowadays, novel strategies are required to sustainably support plant growth and elicit the biosynthesis of bioactive molecules with functional roles in crops including lettuce. In this work, the polyphenolic profile of lettuce treated with glutamic acid (GA), humic acid (HA), and their combination (GA + HA) was investigated using an untargeted metabolomics phenolic profiling approach based on high-resolution mass spectrometry. Both aerial and root organ parts were considered, and a broad and diverse phenolic profile could be highlighted. The phenolic profile included flavonoids (anthocyanins, flavones, flavanols, and flavonols), phenolic acids (both hydroxycinnamics and hydroxybenzoics), low molecular weight phenolics (tyrosol equivalents), lignans and stilbenes. Overall, GA and HA treatments significantly modulated the biosynthesis of flavanols, lignans, low molecular weight phenolics, phenolic acids, and stilbene. Thereafter, antioxidant capacity was evaluated in vitro with 2,2-diphenyln-1-picrylhydrazyl (DPPH), 2,2′-Azino-bis(3-ethylbenzthiazoline-6-sulfonic acid) (ABTS), ferric reducing antioxidant power (FRAP), and cupric ion reducing antioxidant capacity (CUPRAC) assays. In addition, this study examined the inhibitory properties of enzymes, including acetylcholinesterase (AChE) and butyrylcholinesterase (BChE), tyrosinase, alpha-amylase, and alpha-glucosidase. Compared to individual treatments, the combination of GA + HA showed stronger antioxidant abilities in free radical scavenging and reducing power assays in root samples. Moreover, this combination positively influenced the inhibitory effects of root samples on AChE and BChE and the tyrosinase inhibitory effect of leaf samples. Concerning Pearson’s correlations, antioxidant and enzyme inhibition activities were related to phenolic compounds, and lignans in particular correlated with radical scavenging activities. Overall, the tested elicitors could offer promising insights for enhancing the functional properties of lettuce in agricultural treatments.

## 1. Introduction

Lettuce (*Lactuca sativa* L., Asteraceae) is a widely grown and popular vegetable, especially consumed in the human diet [1,2]. A diet rich in fruits and vegetables promotes physical and mental health, prevents disease, and increases the quality of life. Researchers use this strategy to explore natural plant species that can control various metabolic risk factors and identify bioactive compounds, such as polyphenols, carotenoids, and vitamins, to restrain obesity development [1], as well as lower the risk of chronic diseases [3]. *L. sativa* contains a diversity of antioxidant compounds, including phenolics, carotenoids, and ascorbic acid [4]. The total phenolic contents may vary depending on cultivar type, leaf pigmentation, and growth stage [5,6]. For example, a previous study reported a higher total phenolic content in red-leaf lettuces than the popularly used green-leaf lettuce [7]. The reason for its red color may be caused by anthocyanins, which are a subgroup of phenolic contents. However, the demand for nutritious vegetables such as lettuce is expected to increase by 60% by 2050 due to the increasing world population [8]. In addition, it is reported that production should be increased by 70% to sustain the growing population [9]. Based on all these approaches, novel strategies are required to develop or accelerate the biosynthesis of molecules related to cell structures and to provide more information about the functional food components and bioactive roles of lettuce. During its cultivation, lettuce needs fertilizers and pesticides intensely [10]. The adequate supply of fertilizers and pesticides alters the properties and functions of cultivated soils, such as the activities of soil enzymes, moisture, rhizodeposition, organic carbon, nutrient content, and pH [11]. However, the misutilization of synthetic fertilizers can lead to contamination of the agroecosystem. To improve both sustainability of ecosystems and plant productivity, organic matter is used as an agronomic strategy for decreasing the risks of soil degradation and protecting soil quality. The decomposition of natural organic matter brings forth humic substances, which have positive roles in the promotion of growth and yield of plants, as well as improved adsorption of nutrients and enzyme activities related to primary and secondary metabolisms [12]. Humic acid (HA) is a water-soluble organic acid naturally included in soil, peat, and lignin [13]. As an organic fertilizer, HA can markedly affect the optimum growth of plants and can induce the efficient use of K, N, Mg, Ca, and P elements [14]. It can also adjust soil structure, such as pH, anion–cation balance, and affect cell respiration, protein synthesis, water uptake, photosynthesis, and enzyme activity [15]. All these features of humic acid would improve the yield of the product, as well as their quantity and quality. In this sense, several studies have been performed on the effect of humic acid on the yield of different plants [16,17,18,19,20,21], but little information is available about the interaction between HA and bioactive compounds and the effects of these interactions on physiological or biochemical processes.

In addition to their biostimulant function, protein hydrolysate products are a rich source of amino acids and provide an alternative contribution to improving the agronomic performance of plants without resorting to genetic alterations [22]. Amino acids can impact seed germination, osmotic adjustment, fruit maturation, and activation of systems in response to stresses [23]. L-glutamic acid (GA), a non-essential amino acid, acts as a signaling molecule in growth and development systems and as a defense against adverse conditions [24,25]. In addition, GA is shown to be involved in some essential biological processes, such as scavenging reactive oxygen species (ROS) [26], chlorophyll synthesis [27], controlling stomatal movement [28], and tricarboxylic acid cycle (TCA) metabolism [29].

Up to the present, there have been several findings about the responses of the individual treatments of HA or GA on plants [26,30]. However, the antagonistic/synergistic impacts that could result from the mixture of HA and GA are unknown. In addition, the combined effect both or even alone is infrequently examined under hydroponic conditions [31,32]. To improve our knowledge, it is indispensable to understand what changes the exogenously applied HA and GA, individually or in combination, play on the physiologic and metabolic processes of *L. sativa*, and the possible interactions including antagonistic, neutral, and synergistic responses between them. Therefore, in the current study, we aimed to determine the effect of HA and GA (individual or combined) on the metabolomic profile and biological activities (antioxidant and enzyme inhibitory) of *L. sativa* parts (leaves and roots). The results obtained from this study could provide new insights for the development of novel foods with enhanced health-promoting properties in agricultural applications.

## 2. Results and Discussion

### 2.1. Total Bioactive Compounds

The TPC and TFC content in *L. sativa* treated with GA, HA, and GA + HA were determined using spectrophotometric methods for both aerial and root organ parts. As can be seen from Table 1, control groups contained the highest TPC in both parts (leaves: 23.68 mg GAE/g; root: 52.43 mg GAE/g). All treatments reduced the TPC in the leaf groups. Similar results were obtained in the root group, although the combined group (GA + HA) contained more phenols than their individual experiments. Different results in terms of TFC have been observed. The TFC in the leaves was increased with the HA application (52.44 mg RE/g) compared to the control group (49.33 mg RE/g). However, in the leaves group, the total flavonoid content was reduced in the GA and combined group. Regarding the root extracts, the level was similar in the control and combined groups but was reduced in the individual GA and HA groups. In a previous study by Muscolo et al. [33], HA application had a negative effect on TPC. In contrast, it has been reported by several researchers that HA was a positive modulator of total phenolic levels in some plants [13,34,35]. The conflicting results could be explained by the different HA concentrations and different plants used. While spectrophotometric assays are commonly used for analyzing total phenolic content, they may not provide accurate measurements of these compounds, as other phytochemicals, such as proteins, can also react with the Folin–Ciocalteu reagent [36]. Therefore, further confirmation of the results is required using chromatographic techniques, such as HPLC or HPLC-MS.

### 2.2. Metabolomic Profiling of Lettuce Treated with Glutamic Acid, Humic Acid, and Their Combination

The effect of GA, HA, and their combination (GA + HA) on the modulation of phenolic compound production on lettuce, considering both the effect on leaves and roots, was investigated using an untargeted metabolomics approach. The phenolic profile allowed us to record 423 features, characterized mainly by flavonoids, as the most frequent class of phenolic compound, with 207 features (i.e., 45 anthocyanins, 10 flavanols, and 152 other flavonols), followed by tyrosol and low molecular weight phenolic (LMW) equivalents (81 metabolites), phenolic acids (96 metabolites), lignans (29 metabolites), and stilbenes (10 metabolites). The whole list of polyphenols annotated is provided in the Supplementary Materials (Table S1), comprehensive of their classification (class and subclass), abundances, retention time, and mass spectrum.

**Figure 1:**
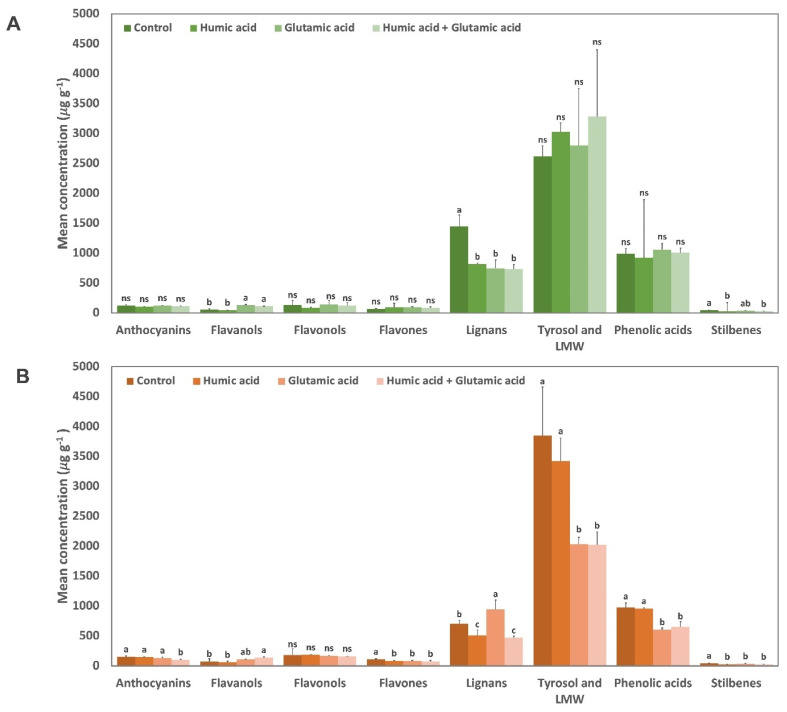
Semi-quantitative analysis of different phenolic subclasses in lettuce organ parts: (**A**) leaves and (**B**) roots. Values are expressed as the mean concentration (µg g^−1^ dry matter) from three replicates. The letters in the same phenolic subclass indicate significant differences between treatments (ANOVA and Tukey’s HSD post hoc test, *p*-value < 0.05). Abbreviations: LMW: Low-molecular-weight phenolic compounds; ns: no significative.

A semi-quantitative analysis of the main phenolic classes was carried out to assess the effect of different treatments on lettuce organ parts, expressed as µg phenolic equivalents g^−1^ dry matter (DM), and reported in Figure 1A,B, as leaf and root extracts, respectively. Moreover, semi-quantitative values are reported in Supplementary Table S2. Overall, the phenolic classes most abundant in extracts from the leaves and roots of lettuce were represented by lignans, tyrosols and LMW, and phenolic acids. However, the application of GA, HA, and GA + HA heavily affected their modulation, reported mostly on root organ parts than on leaves. This behavior suggests that the application of these compounds had a nutritional and plant development function. The positive effects of GA on the plant’s growth were described in the work published by Liao et al. [37]. Glutamic acid is one of the most important amino acids that can stimulate both primary and secondary metabolism, improving photosynthetic activity and leaf functionality [29,38,39]. Glutamic acids and HA were reported by Haghighi et al. [40] as being good organic N sources able to increase plant yields and nutrient adsorption. Considering the aerial part, the use of either GA or HA significantly increased flavone content, to 94.43 and 99.49 µg Eq. g^−1^, respectively (Table S2). The most modulated flavones were luteolin, 6-hydroxyluteolin, hispidulin, nobiletin, and tetramethylscutellarein. Compared to the control, the flavanols content was increased after GA and GA + HA treatments and were represented by (+)-catechin and (−)-epicatechin. In contrast, all treatments led to a decrease in lignans and stilbenes in leaf tissues (Figure 1A). The most heavily affected lignan compounds were anhydro-secoisolariciresinol, episesaminol, secoisolariciresinol-sesquilignan, sesaminol, sesamol, and sesamolin. Lignans and stilbenes are compounds generally associated with a response induced by biotic or abiotic stresses. In previous works [41,42], the data showed increased lignans and stilbenes in response to treatment with biostimulants under stress conditions, suggesting a clear elicitation of their biosynthesis under a stress response. However, in the absence of stress factors, the metabolism of polyphenols could be pushed towards the accumulation of different classes of compounds (flavanols and flavones) not directly involved in a plant’s defense mechanism. Accordingly, treatment with GA and GA + HA resulted in a significant accumulation of flavanols. Similar results were highlighted by Savarese et al. [43], where the use of HA in combination with a microbial inoculum on lettuce significantly affected both primary and secondary metabolism, increasing the biosynthesis of essential amino acids, carbohydrates, and polyphenolic compounds.

Different results occurred when the root organ parts were considered (Figure 1B). Indeed, the application of GA, HA, and GA + HA resulted in a significant variation in the content of flavones (*p* < 0.05), LMW (*p* < 0.01), phenolic acid (*p* < 0.001), stilbenes (*p* < 0.01), and lignans (*p* < 0.01) (Table S2). Root extracts treated with HA significantly reduced the accumulation of apigenin diglucoside (as flavones) and episesamin and sesamin (as lignans), while GA application resulted in a positive modulation of lignans such as lariciresinol-sesquilignan, secoisolariciresinol-sesquilignan, medioresinol, and trachelogenin. Moreover, the latter negatively modulated the accumulation of flavones (luteolin 7-O-(2-apiosyl-glucoside), chrysoeriol 7-O-(6″-malonyl-glucoside), diosmin, and neodiosmin), LMW (i.e., oleuropein, 4-vinylguaiacol, and 3-Methoxyacetophenone), phenolic acids (i.e., 5-O-galloylquinic acid, 1,2,2′-triferuloylgentiobiose, and ellagic acid arabinoside) and stilbenes (pinosylvin and piceatannol 3-O-glucoside) on root extracts (Table S1). Interestingly, the combined use of GA and HA resulted in an overall reduction in phenolic compound accumulation as observed under GA treatment, with the expectation of flavonols and flavanols. Indeed, (+)-catechin and (−)-epicatechin as the class of flavanols were significantly increased after GA + HA treatment. The use of HA and amino acids is known to increase plant yields and influence root absorption of nutrients, such as phosphorus, nitrogen, and potassium, as well as the biosynthesis of phenolic compounds involved in antioxidant activity [44]. Polyphenols also play an important role in a plant’s physiological modulation, including inhibitors of the auxin transport system, regulation effect of nutrients uptake, or root architecture [45,46]. Indeed, it has been reported that plant phenolic compounds, such as flavonoids and lignin precursors, accumulate in response to environmental stresses and are considered crucial defense compounds that can eliminate harmful ROS. However, these secondary metabolites could be down-modulated under optimum nutrient conduction to activate energies and resources for plant development [47].

### 2.3. Multivariate Discrimination Analysis of Lettuce Treated with Glutamic Acid, Humic Acid, and Their Combination

**Figure 2:**
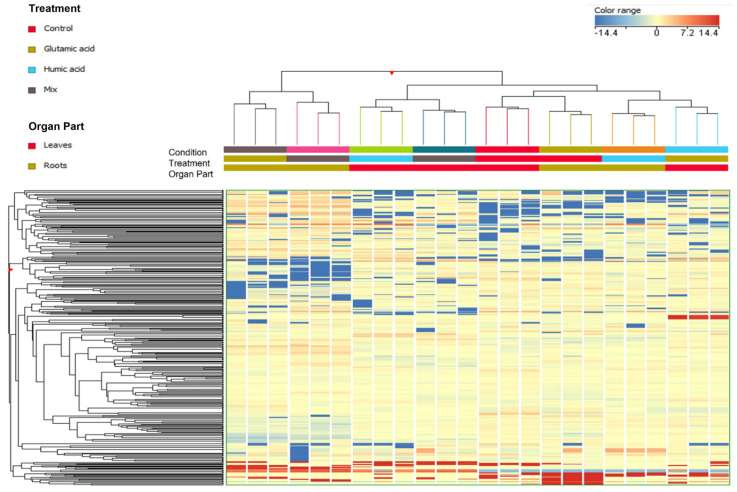
Unsupervised hierarchical clustering analysis (HCA, Euclidean distance) performed using Log2 fold change median normalized values for each annotated compound in lettuce leaves and roots extract treated with Glutamic acid, Humic acid, and their combination (mix).

The discrimination analysis was carried out using two different approaches. The first approach consists of an unsupervised hierarchical cluster analysis (HCA) that clusters samples based on their similarities and/or dissimilarities in the phenolic profiles among different treatments (Figure 2). As reported in the figure, the HCA identified four different clusters highlighting the major effect of the treatments on the phenolic profile compared to the plant tissue. Indeed, the first main cluster was distinctly characterized by the effect of GA and GA + HA on the modulation of the phenolic profile of the root extract, which was found to have a different profile from the other extracts. In contrast, the second group highlights the effect of HA and GA + HA on leaf phenolic modulation. Both clusters emphasize the different performance of GA and HA and GA + HA on the lettuce organ part. Finally, the third and fourth clusters consisted of untreated matrices and those treated with HA and GA on the roots and leaves of lettuce, respectively. The latter two clusters suggested less modulation potential of the considered treatments since they were similar to the untreated control (Figure 2).

**Figure 3:**
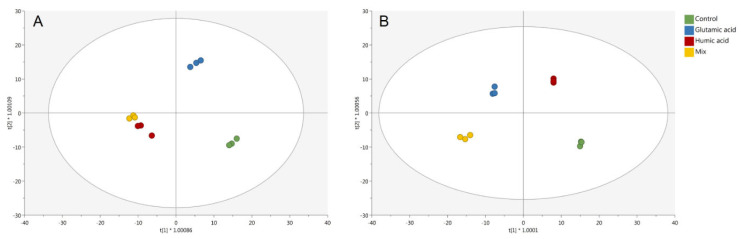
Supervised orthogonal projections to latent structures discriminant analysis (OPLS-DA) score plot of lettuce leaves (**A**) and roots (**B**) built according to the phenolics profile following application of glutamic acid, humic acid, and their combination (mix).

The second approach adopted was the supervised Orthogonal Projection to Latent Structure Discriminant Analysis (OPLS-DA; Figure 3). Figure 3 reports the OPLS-DA score plot for leaves (Figure 3A) and roots (Figure 3B) organs, respectively. The OPLS-DA model generated for leaf tissue confirmed the data produced by HCA, reporting high discrimination performance between HA and GA + HA treatment compared to control and GA treatment on leaf extract (Figure 3A). The score plot generated was characterized by high performance parameters, such as goodness of fit (R^2^) and the prediction capacity of this model (Q^2^) values as 0.908 and 0.989, respectively. Moreover, the model was validated through cross-validation (CV-ANOVA *p*-value < 0.05) and the permutation tests to exclude model overfitting.

The variable importance in projection (VIP) compounds prioritization approach was used to identify the most discriminant compounds contributing to the differences outlined in the OPLS-DA model, using a VIP score ≥ 1.2. VIP markers are provided in Table 2, including compound classification, VIP score ± standard errors, and log fold change obtained using pairwise comparison between treatments and control. As provided, the compounds with high discrimination potential were hydroxycinnamic acids (3,4-diferuloylquinic acid and ellagic acid acetyl-xyloside), followed by anthocyanins (malvidin 3-O-galactoside), and flavonols (quercetin 3-O-(6-malonyl-glucoside)). These compounds were mainly accumulated on GA- and HA-treated leaves and decreased under GA + HA treatments. Conversely, the metabolites that mostly accumulated after GA + HA treatments were tyrosols and LMW phenolics (4-vinylsyringol, phlorin, isopimpinellin, and 5-pentadecylresorcinol), followed by flavones (nobiletin) and phenolic acids (sinapic acid).

The OPLS-DA model generated for root organ parts could discriminate all four treatments with the two latent vectors. Indeed, the score plot generated was characterized by high-performance parameters (R^2^ = 0.995 and Q^2^ = 0.93) and without model overfitting. The list of VIP biomarkers (VIP ≥ 1.2) extrapolated from the model is provided in Table 3, including the compound classification, VIP score ± standard errors, and log fold change obtained using a pairwise comparison between treatments and the control. The treatment with GA on the root organ parts positively modulated the accumulation of myricetin, pelargonidin 3-O-arabinoside (flavonoids), ellagic acid acetyl-xyloside, 1,2-disinapoylgentiobiose (phenolic acids), medioresinol (lignans), and eugenol (LMW). Conversely, 5-5′-dehydrodiferulic acid and p-Coumaroyl tyrosine (phenolic acids) were decreased after GA treatment, with LogFC −4.16 and −4, respectively. Similar to the GA treatment, HA application produced a high accumulation of ellagic acid acetyl-xyloside, followed by petunidin 3-O-(6″-acetyl-glucoside), myricetin (flavonoids), dihydrocaffeic acid, and 1,2-disinapoylgentiobiose (phenolic acids). Finally, the combination of GA and HA application resulted in an accumulation of Petunidin 3-O-(6″-acetyl-glucoside) (anthocyanins), Medioresinol (lignans), and 3-Sinapoylquinic acid (phenolic acid).

### 2.4. Antioxidant Properties

The antioxidant properties of the tested groups were examined using complementary in vitro methods. DPPH and ABTS radicals are commonly used to evaluate the radical scavenging properties of plant extracts. In the DPPH assay, the best scavenging ability was found in the control group. The application of humic and glutamic acid resulted in a dramatically reduced scavenging ability. In roots, the combined group (62.08 mg TE/g) had increased DPPH scavenging ability compared to the individual glutamic acid (47.07 mg TE/g) and humic acid (46.36 mg TE/g) treatments (Table 1). In general, similar results were found in the ABTS assay, and the combined group was more active in both plant parts than the single applications. The reducing power is an important indicator for assessing the electron-donating ability of antioxidants. To this end, we studied the conversion of Cu^2+^ to Cu^+^ and Fe^3+^ to Fe^2+^ in CUPRAC and FRAP assays, respectively. Similar to the free radical scavenging assays, the reducing ability was reduced by applying humic and glutamic acid and their combination compared to the control groups. The observed radical scavenging and reducing power abilities can be explained by the decreased phenolic contents in the application groups. Total antioxidant capacity was also determined using a phosphomolybdenum assay, and the control groups had the highest ability in both plant parts (leaves: 0.66 mmol TE/g; roots: 1.02 mmol TE/g) (Table 1). Furthermore, the combined application (0.93 mmol TE/g) in the root groups had a higher overall antioxidant capacity compared to individual glutamic acids (0.80 mmol TE/g) and humic acids (0.90 mmol TE/g). Regarding metal chelating abilities, in the leaf groups, all tested groups showed similar metal chelating ability. However, all applications positively affected the metal chelating ability in the root groups. In particular, the humic acid application showed the highest metal chelating ability (63.63 mg EDTAE/g) (Table 1). In summary, the applications did not affect the antioxidant properties that much. Different results can be found in the literature for different plants. Humic acids have been considered effective elicitors for some plants [13,35,48]. In contrast, the negative effects observed in the presence of elicitors can be attributed to their well-known allelopathic effects, which interfere with photosynthesis, cell division and elongation, membrane fluidity, protein biosynthesis, and the activities of numerous enzymes, resulting in growth failure [49,50,51]. While several antioxidant assays were performed in this study, these assays have some limitations. For instance, DPPH and ABTS are not biological radicals, and their nitrogen centers have steric hindrances, making them poor models for in vivo assays. In the CUPRAC, FRAP, and phosphomolybdenum assays, not only phenolics but also other antioxidants, such as ascorbic acid and tocopherols, can affect the observed transformations in the assays. In addition, the reaction of phosphomolybdenum takes a long time to complete. Regarding the metal chelating assay, the assay is not specific, and several compounds including polysaccharides and sulfides can be effective [52]. Taken together, these assays provide an initial insight into the potential of plant extracts, and further confirmation of the results using in vivo assays is strongly recommended.

### 2.5. Enzyme Inhibitory Effects

Enzyme inhibitors are cornerstones in pharmaceutical and nutraceutical applications. Several diseases could be treated by inhibiting key enzymes, including Alzheimer’s disease, obesity, and type II diabetes. In this sense, applications that increase enzyme inhibitory properties are gaining interest. In the current study, we tested the inhibitory effects of the different groups against cholinesterase, tyrosinase, amylase, and glucosidase. The results are shown in Table 4. For AChE inhibition, all samples showed similar abilities in the leaf groups. Regarding root AChE inhibition, the combined group was positively affected by the inhibitory properties compared to the control. For BChE inhibition, the combined groups were more active for both parts than controls and single applications. Based on these results, combining glutamic and humic acids could be valuable for treating or managing Alzheimer’s disease. Consistent with our results, Akincioglu et al. [53] reported significant cholinesterase inhibitory effects when using humic acid. Regarding tyrosinase inhibitory properties, especially in leaves, all treatments were significantly increased, and the highest level of ability was obtained in the combined group. In root samples, the ability was increased with glutamic acid and the combined groups, but it was not statistically important (*p* > 0.05). Inhibition of amylase and glucosidase is related to antidiabetic properties. In both parts, amylase inhibition was reduced with all experimental groups. However, except for the application of humic acid to roots, other applications positively affected glucosidase inhibitory properties compared to the control groups (Table 4). In general, using the combined group improved the enzyme-inhibiting properties. Several applications of the enzyme-inhibiting properties of some plants have been raised in the literature [54,55,56]. In this sense, combining glutamic acid and humic acids could be useful for developing functional products enriched with enzyme-inhibiting properties.

### 2.6. Pearson’s Correlation

Pearson’s coefficients were calculated to highlight the possible correlations between bioactive compounds and biological activities reported for lettuce leaf and root extracts (Supplementary Table S3). Phenolic compounds derived from lettuce leaf extracts reported a high correlation with antioxidant and enzyme inhibition activities. Interestingly, lignan compounds showed a high positive correlation with DPPH, ABTS, CUPRAC, and phosphomolybdenum activities (r > 0.7; *p* < 0.01) and a negative correlation with tyrosinase and a-glucosidase activities (r < −0.84; *p* < 0.01). However, stilbenes showed a positive correlation with DPPH (r = 0.729; *p* < 0.01) and a negative correlation with AChE, tyrosinase, and a-glucosidase (r = −7.95, r = −7.03, and r = −7.16, respectively). Flavanols extracted from lettuce leaves showed a negative correlation with antioxidant activities (CUPRAC, FRAP, and phosphomolybdenum) with correlation coefficients greater than 0.778 (*p* < 0.01). Considering the root extracts, the pool of phenolics and flavonoids was reported to be positively correlated with antioxidant activities and inhibition activity against a-glucosidase (r > 0.7; *p* < 0.01) and negatively correlated with metal chelating activity and inhibition against BChE. Moreover, anthocyanins and LMW phenolic classes positively correlated with a-amylase. The relationship between antioxidant activity and lignans and stilbenes is well described in the literature [57,58,59]. The scavenging activity of lignans and stilbenes depends on their structural features. According to the literature, a decreased quantification of lignans and stilbenes is closely related to decreased antioxidant activity assays. Kose et al. [59] tested the lignan antioxidant activity under different conditions and, in particular, showed that lignans had the ability to generate stable products by giving electrons to free radicals and ROS. Soleymani et al. [60] related the decrease in sasaminol, sesamal, and sesamolin to a decrease in antioxidant activity, and they tested this ability under different conditions. Lignans, stilbenes, and flavonoids are correlated to a different modulation of the enzyme inhibitor effects, particularly the inhibition of the α-glucosidase enzyme [61,62]. Regarding the inhibition activities, related to this class of compounds, the accepted mechanism of action regards the capability to maintain enzymes in their reduced states.

## 3. Materials and Methods

### 3.1. Plant Material and Experimental Design

During the sterilization process of lettuce seeds (*L. sativa* L.), 5% sodium hypochlorite was used for 10 min and the seeds were germinated. Seven-day-old lettuce plants were grown in 1/4 Hoagland solution including 1 mM KNO_3_, 1 M CaNO_3_, 0.4 M MgSO_4_, 0.2 M KH_2_PO_4_, 0.03 M H_3_BO_3_, 0.002 M CuSO_4_, 0.004 M ZnSO_4_, 0.005 M MnCl_2_, 0.001 M (NH_4_)Mo_7_O_24_, and 0.2 M Fe-EDTA under controlled conditions for fourteen days. The solution was refreshed every 5 days. Humic acid (100 mg L^−1^) and glutamic acid (100 mg L^−1^) were applied individually and in combination, in addition to the growth medium. These concentrations were determined based on the results observed by Li et al. [63] and Kaya et al. [64]. The experiment was performed using a fully randomized design with three replications.

### 3.2. Untargeted Phenolic Compounds Profiling Using UHPLC QTOF-MS Spectrometry

Lettuce leaf and root samples were extracted using a mechanical homogenizer (Polytron PT 1200 E, Kinematica AG, Malters, Switzerland) in a hydroalcoholic solution (80% methanol acidified with 0.1% formic acid *v*/*v*) using a dilution factor of 1:10. After the homogenization, samples were centrifugated for 10 min at 6000× *g* in a refrigerated centrifuge (Eppendorf 5810R, Hamburg, Germany), and the supernatants were collected and filtrated trough 0.22 µm cellulose filters in amber vials. For untargeted phenolic profiling, samples were analyzed with ultrahigh-pressure liquid chromatography coupled to a quadrupole time-of-flight mass spectrometry (UHPLC-ESI/QTOF-MS; Agilent Technologies, Santa Clara, CA, USA). The analytical condition was reported in our previous work [65]. The chromatographic separation was achieved in the reverse phase using a C18 column (Agilent Zorback Eclipse Plus 4.6 mm × 150 mm, nominal surface area of 160 m^2^/g, and controlled pore size of 95 Å) and water–acetonitrile gradient elution (6–94% in 33 min). The positive full scan mode was used to acquire accurate mass in the 100–1200 *m*/*z* range at 0.8 spectra/s. The injection volume was 6 μL considering three replicates for each sample. After data acquisition, compound identifications were conducted using the software Profinder B.08 (from Agilent Technologies, Santa Clara, CA, USA), according to the ‘find-by-formula’ algorithm against the Phenol-explorer 3.6 database [66]. The annotation workflow allowed compound identification according to Level 2 confidence (i.e., putatively annotated compounds), concerning the COSMOS Metabolomics Standards Initiative [67]. Afterward, cumulative intensities per class of compounds were calculated, and qualitative concentrations were determined using calibration curves of pure individual standard compounds (expressed as mg equivalents/g dry weight (dw); Extrasynthese, Lyon, France; purity > 98%) analyzed under the same conditions: ferulic acid (phenolic acids), quercetin (flavonols), sesamin (lignans), cyanidin (anthocyanins), catechin (flavan-3-ols), luteolin (flavones and other remaining flavonoids), resveratrol (stilbenes), and tyrosol (tyrosols and other polyphenols).

### 3.3. Profile of Bioactive Compounds

To determine the total phenolic and flavonoid contents, we utilized Folin–Ciocalteu and AlCl3 assays, respectively [68]. The results were expressed as milligrams of gallic acid equivalents per gram of extract (mg GAE/g) for total phenolic content and as rutin equivalents for total flavonoid content (mg REs/g extract).

### 3.4. Determination of Antioxidant and Enzyme Inhibitory Effects

The extracts were evaluated for antioxidant and enzyme inhibitory activity using established methods [69,70]. For DPPH and ABTS radical scavenging, the CUPRAC and FRAP assays were expressed as mg Trolox equivalents (TE)/g extract. Metal chelating ability (MCA) was reported as mg EDTA equivalents (EDTAE)/g extract, while the total antioxidant activity (phosphomolybdenum assay, PBD) was expressed as mmol TE/g extract. AChE and BChE inhibitory activities were given as mg galanthamine equivalents (GALAE)/g extract, tyrosinase inhibitory activity was expressed as mg kojic acid equivalents (KAE)/g extract, and amylase and glucosidase inhibitory activities were presented as mmol acarbose equivalents (ACAE)/g extract.

### 3.5. Statistical Analysis

A one-way analysis of the variance (ANOVA with Tukey’s post hoc test) was performed using data from each assay and using XlStat 16.0 software. Pearson correlations (*p* < 0.05; two-tailed) were carried out using the software PASW Statistics 26.0 (SPSS Inc., Chicago, IL, USA).

The chemometrics analysis was conducted using Mass Profiler Professional B12.6 (Agilent Technologies, Santa Clara, CA, USA), and data normalization was performed according to previously published work [71]. Specifically, identified compounds were filtered by frequency, normalized at the 75th percentile, and baselined to the median of all samples. The resulting dataset was investigated using unsupervised hierarchical cluster analysis (HCA) and supervised Orthogonal Projections to Latent Structures Discriminant Analysis (OPLS-DA; SIMCA 16, Umetrics, Malmo, Sweden) approaches. The OPLS-DA models for the leaves and roots were successively cross-validated (CV-ANOVA; *p* < 0.01), and permutation testing was completed after inspecting model parameters (goodness-of-fit R^2^Y and goodness-of-prediction Q^2^Y) and model outliers (Hotelling’s T2). The variable importance in projection (VIP) metabolites, having a score >1.2, were extrapolated as biomarkers responsible for the discrimination capacity of the models. In addition, a fold-change (FC) analysis was performed to provide the up/down-accumulation trends in each discriminant marker arising from the VIP selection methods.

## 4. Conclusions

This study demonstrates the impact of glutamic and humic acid application, individually and in combination, on the metabolomic profile and biological abilities of lettuce. The combined application of these elicitors significantly enhanced biological abilities, such as free radical scavenging and cholinesterase inhibition. Metabolomic analysis revealed that the application of these elicitors modulated the synthesis of biologically active compounds, including flavonoids, phenolic acids, and stilbenes. These findings could aid in the production of lettuce with improved functional properties for agricultural and nutraceutical applications.

## Figures and Tables

**Table 1:** Total bioactive compounds and antioxidant properties of the tested groups.

Part; Treatment	TPC (mg GAE/g)	TFC (mg RE/g)	DPPH (mg TE/g)	ABTS (mg TE/g)	CUPRAC (mg TE/g)	FRAP (mg TE/g)	PBD (mmol TE/g)	MCA (mg EDTAE/g)
Leaves; Control	23.68 ± 0.75 ^a^	49.33 ± 1.95 ^ab^	9.97 ± 0.54 ^a^	35.59 ± 1.88 ^a^	71.59 ± 0.35 ^a^	29.52 ± 0.47 ^a^	0.66 ± 0.01 ^a^	16.60 ± 3.39 ^a^
Leaves; Humic acid	22.48 ± 0.43 ^b^	52.44 ± 1.42 ^a^	2.67 ± 0.26 ^b^	24.18 ± 1.85 ^bc^	65.71 ± 1.01 ^b^	30.55 ± 0.42 ^a^	0.61 ± 0.02 ^b^	14.87 ± 3.40 ^a^
Leaves; Glutamic acid	20.32 ± 0.40 ^c^	47.50 ± 0.15 ^bc^	1.35 ± 0.20 ^c^	20.33 ± 1.05 ^c^	55.58 ± 0.97 ^d^	23.54 ± 0.21 ^c^	0.56 ± 0.01 ^bc^	13.09 ± 2.94 ^a^
Leaves; Humic acid + glutamic acid	20.58 ± 0.35 ^c^	45.03 ± 0.88 ^c^	1.42 ± 0.09 ^c^	26.57 ± 0.82 ^b^	58.55 ± 1.14 ^c^	26.06 ± 1.15 ^b^	0.55 ± 0.03 ^c^	13.68 ± 0.32 ^a^
Roots; Control	52.43 ± 0.47 ^a^	7.13 ± 0.47 ^a^	66.38 ± 0.50 ^a^	85.03 ± 3.32 ^a^	188.20 ± 1.36 ^a^	74.91 ± 0.97 ^a^	1.02 ± 0.05 ^a^	50.39 ± 3.78 ^b^
Roots; Humic acid	39.70 ± 0.29 ^d^	4.41 ± 0.36 ^b^	46.36 ± 1.00 ^c^	62.86 ± 1.84 ^c^	138.94 ± 0.23 ^c^	58.29 ± 1.78 ^b^	0.90 ± 0.05 ^bc^	63.63 ± 5.33 ^a^
Roots; Glutamic acid	44.52 ± 0.56 ^c^	5.26 ± 0.36 ^b^	47.07 ± 0.81 ^c^	68.05 ± 2.77 ^c^	142.39 ± 2.46 ^c^	59.99 ± 0.70 ^b^	0.80 ± 0.03 ^c^	55.04 ± 0.23 ^ab^
Roots; Humic acid + glutamic acid	48.96 ± 0.26 ^b^	7.39 ± 0.39 ^a^	62.08 ± 2.05 ^b^	77.06 ± 0.50 ^b^	174.83 ± 2.41 ^b^	72.59 ± 0.34 ^a^	0.93 ± 0.04 ^ab^	56.26 ± 2.90 ^ab^

Values are reported as mean ± SD of three parallel measurements (*n* = 3). GAE: Gallic acid equivalent; RE: Rutin equivalent; TE: Trolox equivalent; EDTAE: EDTA equivalent; ABTS, 2,2′-azino-bis(3-ethylbenzothiazoline) 6-sulfonic acid; CUPRAC, cupric ion reducing antioxidant capacity; DPPH, 1,1-diphenyl-2-picrylhydrazyl; EDTAE, EDTA equivalents; FRAP, ferric ion reducing antioxidant power; MCA: Metal chelating ability; PBD: Phosphomolybdenum; TPC: Total polyphenol content; TFC: Total flavonoid content. Different superscript letters indicate significant differences in the tested group for each part (from ANOVA, Tukey’s post hoc test, *p* < 0.05).

**Table 2:** Variable importance in projection (VIP) markers identified in lettuce leaves for the discrimination of glutamic acid (GA), humic acid (HA), and their combination (GA + HA) application. Discriminant phenolic compounds are provided with their compound classification, VIP scores ± standard errors (VIP ≥ 1.20), and log fold change values obtained from the pairwise comparison between different treatments and the control.

Primary ID	Class	Sub Class	VIP Score ± SE	Log FC [GA] vs. [C]	Log FC [HA] vs. [C]	Log FC [GA + HA] vs. [C]
Malvidin 3-O-galactoside	Flavonoids	Anthocyanins	1.30 ± 0.34	1.08	0.81	−0.57
Cyanidin 3-O-arabinoside			1.29 ± 0.42	−4.00	1.09	−4.00
Malvidin 3,5-O-diglucoside			1.26 ± 0.28	−4.00	−1.68	−0.47
Phloridzin		Dihydrochalcones	1.23 ± 0.29	−0.4	0.06	−0.18
(+)-Catechin		Flavanols	1.25 ± 0.20	1.44	−0.21	1.30
Hesperetin		Flavanones	1.30 ± 0.38	−1.62	0.67	−1.34
Jaceosidin		Flavones	1.29 ± 0.29	0.54	0.40	−0.22
Luteolin 7-O-glucuronide			1.25 ± 0.49	1.28	0.71	−0.89
Nobiletin			1.22 ± 0.30	−0.61	4.88	3.71
Quercetin 3-O-(6″-malonyl-glucoside)		Flavonols	1.30 ± 0.35	1.13	0.82	−0.58
Quercetin 3-O-glucuronide			1.28 ± 0.40	1.03	0.66	−0.33
Quercetin 3-O-glucoside			1.26 ± 0.46	0.07	−0.1	−1.18
Quercetin 3-O-arabinoside			1.25 ± 0.28	0.96	−4.00	1.16
6″-O-Malonyldaidzin		Isoflavonoids	1.21 ± 0.48	4.00	-	-
7-Oxomatairesinol	Lignans	Lignans	1.28 ± 0.40	0.66	2.70	1.12
4-Vinylsyringol	Other polyphenols	Alkylmethoxyphenols	1.25 ± 0.30	2.57	2.59	5.06
5-Pentadecylresorcinol		Alkylphenols	1.25 ± 0.30	−1.03	−1.05	1.31
5-Nonadecylresorcinol			1.22 ± 0.49	−0.54	−1.48	−0.47
Demethoxycurcumin		Curcuminoids	1.26 ± 0.18	0.68	−0.33	0.46
Isopimpinellin		Furanocoumarins	1.23 ± 0.30	1.93	0.05	1.79
4-Hydroxycoumarin		Hydroxycoumarins	1.21 ± 0.54	2.25	1.35	0.08
Phlorin		Other polyphenols	1.28 ± 0.33	−0.28	−0.39	3.10
Ellagic acid acetyl-xyloside	Phenolic acids	Hydroxybenzoic acids	1.31 ± 0.29	2.59	2.21	−4.00
4-Hydroxybenzoic acid 4-O-glucoside			1.26 ± 0.42	-	0.93	−0.18
3,4-Diferuloylquinic acid		Hydroxycinnamic acids	1.33 ± 0.22	3.33	3.81	−0.35
Chicoric acid			1.29 ± 0.28	3.7	2.99	0.26
3,4-Dicaffeoylquinic acid			1.25 ± 0.4	−0.95	2.34	0.22
3-Caffeoylquinic acid			1.23 ± 0.33	2.49	0.40	0.93
Sinapic acid			1.22 ± 0.33	1.80	−0.05	1.66
Avenanthramide 2p			1.21 ± 0.30	0.09	−0.81	−0.27

**Table 3:** VIP markers identified in lettuce roots for the discrimination of glutamic acid (GA), humic acid (HA), and their combination (GA + HA) application. Discriminant phenolic compounds are provided with their compound classification, VIP scores ± standard errors (VIP ≥ 1.20), and log fold change values obtained from the pairwise comparison between different treatments and the control.

Primary ID	Class	Sub-Class	VIP Score ± SE	Log FC [GA] vs. [C]	Log FC [HA] vs. [C]	Log FC [GA + HA] vs. [C]
Petunidin 3-O-(6″-acetyl-glucoside)	Flavonoids	Anthocyanins	1.28 ± 0.57	-	4.00	4.75
Pelargonidin 3-O-arabinoside			1.21 ± 0.38	2.90	−0.01	0.51
Hesperidin		Flavanones	1.30 ± 0.44	−0.13	−1.96	1.60
Naringin			1.26 ± 0.32	−1.72	−1.00	0.69
Myricetin		Flavonols	1.20 ± 0.33	5.96	2.70	−1.41
Glycitin		Isoflavonoids	1.23 ± 0.39	−0.68	−0.95	−0.45
Medioresinol	Lignans	Lignans	1.35 ± 0.43	1.72	−0.43	4.00
5-Tricosylresorcinol	Other polyphenols	Alkylphenols	1.21 ± 0.28	−0.67	1.41	−0.05
Eugenol		Hydroxyphenylpropenes	1.28 ± 0.71	5.84	1.04	0.44
3,4-DHPEA-EA		Tyrosols	1.27 ± 0.46	−2.07	−0.78	−0.09
p-HPEA-EA			1.20 ± 0.34	−0.87	0.21	1.08
Ellagic acid acetyl-xyloside	Phenolic acids	Hydroxybenzoic acids	1.28 ± 0.47	4.00	4.00	−0.75
Protocatechuic acid 4-O-glucoside			1.23 ± 0.33	−1.58	−1.92	0.35
p-Coumaroylquinic acid		Hydroxycinnamic acids	1.27 ± 0.20	−0.58	−0.26	−0.13
1,2-Disinapoylgentiobiose			1.26 ± 0.34	1.14	1.52	−0.68
Caffeic acid			1.26 ± 0.18	−0.23	−0.19	0.94
5-5′-Dehydrodiferulic acid			1.25 ± 0.31	−4.16	−0.45	−0.02
3-Sinapoylquinic acid			1.23 ± 0.30	−1.29	−2.21	4.00
p-Coumaroyl tyrosine			1.22 ± 0.54	−4.00	−1.71	0.6
Dihydrocaffeic acid		Hydroxyphenylpropanoic acids	1.20 ± 0.28	−1.93	1.69	1.61

**Table 4:** Enzyme inhibitory properties of the tested groups.

Part; Treatment	AChE (mg GALAE/g)	BChE (mg GALAE/g)	Tyrosinase (mg KAE/g)	Amylase (mmol ACAE/g)	Glucosidase (mmol ACAE/g)
Leaves; Control	3.45 ± 0.30 ^a^	2.59 ± 0.41 ^b^	38.07 ± 2.73 ^b^	0.71 ± 0.02 ^a^	18.93 ± 1.49 ^b^
Leaves; Humic acid	4.00 ± 0.20 ^a^	1.42 ± 0.12 ^c^	55.44 ± 5.56 ^a^	0.68 ± 0.02 ^ab^	22.41 ± 0.23 ^a^
Leaves; Glutamic acid	3.87 ± 0.14 ^a^	2.60 ± 0.23 ^b^	56.88 ± 4.14 ^a^	0.68 ± 0.01 ^ab^	22.85 ± 0.21 ^a^
Leaves; Humic acid + glutamic acid	3.98 ± 0.34 ^a^	3.37 ± 0.07 ^a^	61.06 ± 3.60 ^a^	0.64 ± 0.01 ^b^	22.82 ± 0.11 ^a^
Roots; Control	3.76 ± 0.24 ^ab^	2.54 ± 0.43 ^c^	75.73 ± 4.67 ^a^	0.75 ± 0.01 ^a^	21.60 ± 0.18 ^a^
Roots; Humic acid	3.72 ± 0.04 ^b^	3.98 ± 0.22 ^a^	73.86 ± 2.95 ^a^	0.74 ± 0.02 ^a^	17.03 ± 0.07 ^b^
Roots; Glutamic acid	3.64 ± 0.03 ^b^	3.73 ± 0.39 ^ab^	81.11 ± 5.68 ^a^	0.72 ± 0.01 ^ab^	21.76 ± 0.33 ^a^
Roots-; Humic acid + glutamic acid	4.06 ± 0.06 ^a^	3.09 ± 0.29 ^bc^	78.97 ± 6.98 ^a^	0.70 ± 0.01 ^b^	22.00 ± 0.02 ^a^

Values are reported as mean ± SD calculated from three parallel measurements (*n* = 3). GALAE: Galanthamine equivalent; KAE: Kojic acid equivalent; ACAE: Acarbose equivalent; AChE, acetylcholinesterase; BChE, butyrylcholinesterase. Different superscript letters indicate significant differences in the tested group for each plant part (from ANOVA, Tukey’s post hoc test, *p* < 0.05).

## Data Availability

Not applicable.

## Supplementary Materials

The following supporting information can be downloaded at: https://www.mdpi.com/article/10.3390/plants12091822/s1, Table S1: Metabolomics dataset on lettuce aerial parts and root extracts annotated with HPLC-QTOF-MS; Table S2: Semi-quantitative analysis of methanolic extracts; Table S3: Pearson’s correlation matrix between different classes of phenolics compounds and biological activities of lettuce aerial parts and root extracts.
