# Supplementary material for: Glutamate, Humic Acids and Their Combination Modulate the Phenolic Profile, Antioxidant Traits, and Enzyme-Inhibition Properties in Lettuce
Source: Plants (Basel). 2023 Apr 28;12(9):1822. doi: 10.3390/plants12091822 (PMC10181196; doi:10.3390/plants12091822)
Supplement: Supplementary file 1 [file plants-12-01822-s001.zip › Table S2.pdf]

Table S2. Semi-quantitative analysis of methanolic extracts obtained from lettuces leaves and roots treated with: standard non-treated, 100 ppm Humic Acid (HA), 100 ppm Glutamic acid (GA), and 100 ppm Humic acid+ 100 ppm Glutamic acid (MIX).

Source of Variance	Anthocyanins µg Eq. g^{-1} DM	Flavanols µg Eq. g^{-1} DM	Flavonols µg Eq. g^{-1} DM	Flavones µg Eq. g^{-1} DM	Lignans µg Eq. g^{-1} DM	LMW µg Eq. g^{-1} DM	Phenolic acids µg Eq. g^{-1} DM	Stilbenes µg Eq. g^{-1} DM
**Control Leaves**	122.20 ± 13.57	53.71 ± 15.86 B	135.19 ± 72.42	67.90 ± 6.29	1446.14 ± 191.61 A	2617.50 ± 178.69	991.13 ± 91.39	44.06 ± 0.88 A
**Humic acid Leaves**	108.99 ± 1.42	44.93 ± 10.23 B	86.86 ± 67.87	94.43 ± 16.88	815.93 ± 148.13 B	3030.22 ± 967.25	926.08 ± 140.53	31.01 ± 1.90 B
**Glutamic acid Leaves**	126.39 ± 0.93	129.04 ± 14.49 A	145.66 ± 62.59	99.49 ± 5.63	747.10 ± 139.49 B	2803.13 ± 951.66	1060.37 ± 105.65	33.94 ± 9.00 AB
**MIX Leaves**	111.39 ± 8.94	110.63 ± 3.15 A	125.85 ± 51.62	85.75 ± 16.65	734.65 ± 76.05 B	3282.76 ± 1116.47	1005.65 ± 78.48	26.06 ± 6.07 B
**Control Roots**	151.48 ± 13.58 a	72.76 ± 45.74 b	180.78 ± 102.99	109.64 ± 9.25 a	703.44 ± 59.38 b	3845.81 ± 812.61 a	973.07 ± 77.83 a	42.67 ± 0.87 a
**Humic acid Roots**	145.13 ± 3.86 a	58.13 ± 20.81 b	182.74 ±5.88	83.78 ± 7.69 b	505.04 ± 93.95 c	3422.15 ± 385.68 a	956.74 ± 15.05 a	26.46 ± 2.25 b
**Glutamic acid Roots**	131.89 ± 12.72 a	105.69 ± 5.86 ab	169.23 ± 0.80	82.81 ± 7.12 b	942.65 ± 155.07 a	2035.36 ± 111.31 b	602.83 ± 30.80 b	33.50 ± 6.71 b
**MIX Roots**	102.08 ± 7.66 b	137.33 ± 12.00 a	160.60 ± 1.42	73.45 ± 15.36 b	466.89 ± 30.51 c	2024.59 ± 214.55 b	652.25 ± 83.59 b	24.59 ± 5.17 b
**Leaves**	ns	***	ns	ns	**	ns	ns	*
**Roots**	*	*	ns	*	**	*	***	*

Data are mean _ standard error; n = 3. Different letters within each column indicate significant differences according to Duncan's multiple range test ($p < 0.05$). The capital letters references to the statistical analysis of the leaves, lowercase letters to the analysis of the roots. The symbols ns, *, **, and *** indicate a nonsignificant or a significant statistical difference at $p < 0.05$, 0.01, and 0.001, respectively
